# Azimuth-controlled multicolor shifting based on subwavelength sinusoidal grating

**DOI:** 10.1515/nanoph-2025-0350

**Published:** 2025-09-24

**Authors:** Tuo Yang, Yihang Zhou, Hongguang Li, Zefeng Rong, Yanyan Huang, Ping Xu, Haixuan Huang, Xia Yuan, Xulin Zhang, Lei Lei, Guijun Li, Yuanyang Wu, Yutong Di, Shuai Geng, Yunpeng Cui, Mengyu Wang, Yuchao Ma, Wenjie Kuang

**Affiliations:** State Key Laboratory of Radio Frequency Heterogeneous Integration, Ministry of Education/Guangdong Province Key Laboratory of Optoelectronic Devices and Systems, College of Physics and Optoelectronic Engineering, 47890 Shenzhen University, Shenzhen 518060, China; State Key Laboratory of Radio Frequency Heterogeneous Integration, Ministry of Education/Guangdong Province Key Laboratory of Optoelectronic Devices and Systems, College of Physics and Optoelectronic Engineering, Shenzhen University, Shenzhen Holoart High Technology Public Limited Company, Shenzhen 518060, China; College of Big Data and Internet, Shenzhen Technology University, Shenzhen 518118, China; School of Automation, Guangdong University of Petrochemical Technology, Maoming 525000, China

**Keywords:** subwavelength grating, sinusoidal grating, structural color, guided mode resonance, anticounterfeiting

## Abstract

Subwavelength grating-based color-shifting devices exhibit dynamically tunable spectral responses under specific resonant conditions, offering advanced applications in optical anticounterfeiting and surface decoration. However, conventional devices remain limited by structural complexity, narrow tuning ranges, low energy efficiency, and high manufacturing costs. Thereby, we propose a design methodology for multicolor shifting devices capable of dynamic color shifts through azimuthal angle rotation (i.e., in-plane rotation of the device). The proposed evaluation function can be adjusted to yield the desired spectral response, enabling diverse color shifts from a single template. Optimization of the geometric parameters and azimuth angles of rectangular gratings using rigorous coupled-wave analysis (RCWA) and an immune algorithm, followed by conversion into a sinusoidal structure to simplify large-area fabrication while maintaining performance. Using this approach, quad- and penta-color shifting devices based on subwavelength sinusoidal gratings were designed, achieving peak reflectance exceeding 45 % for all target colors, with maxima reaching 89 %. Additionally, the quad-color shifting device was fabricated using low-cost exposed holographic interferometry, validating the method. The results simplify the structure compared to conventional subwavelength grating filters, while enhancing optical performance and fabrication feasibility, offering a new approach for cost-effective, high-performance dynamic optical devices.

## 1 Introduction

In nature, iridescent structural colors are commonly observed on the surfaces of organisms such as butterfly wings, bird feathers, and beetle exoskeletons. These colors exhibit angular dependence, resulting from interactions between incident light and periodic nanostructures. Common mechanisms include surface plasmon resonance [1], Mie resonance [2], guided mode resonance (GMR) [3], [4], [5], and Fabry–Pérot multilayer interference [6], [7]. Developing high-performance color filters based on these mechanisms has been the subject of numerous investigations [8], [9], [10], [11], [12] and has been widely adopted in practical optical systems and consumer products.

Optical anticounterfeiting is an important application of structural color filters in the visible light band, where various optical properties are employed to generate visually unique effects that are resistant to decoding or replication. With the widespread use of bi-color shifting technology [13], [14] over the past two decades, the security effectiveness and visual appeal of traditional solutions have waned over time, prompting more innovative alternatives. Quaranta et al. [15] proposed a dual-period rectangular grating composite structure for selective color filtering and redirection of white light. Su et al. [16] demonstrated high-efficiency, narrowband, angle-selective four-color emission using a single-period TiO_2_ metasurface. Wang et al. [17] and Luan et al. [18] proposed orientation-tunable structural colors by designing blazed gratings with tailored layouts, achieving versatile and vivid naked-eye 3D visual effects. Recent advancements in multilayer plasmonic structures [19], grating arrays [20], [21], microlens arrays [22], and other innovative proposals [23], [24] provide increasingly delivered intuitive and visually compelling solutions. For broader, stable deployment, color-shifting devices require a simpler structure, lower upfront costs, quicker production rates, and compatibility with large-scale manufacturing. Accordingly, preserving their desirable visual effects while avoiding reliance on relatively complex fabrication routes, such as electron-beam lithography (EBL), is essential for commercialization.

In recent years, optical variable devices leveraging sinusoidal grating structures for applications such as product anticounterfeiting and sensors have been extensively explored. Hishova et al. [25] enabled coordinated control of zero-order transmission spectra and incident angle via quasi-sinusoidal gratings. Wu et al. [26] achieved mechanically induced color-shifting effects with tunable brightness, hue, and viewing angle using periodic sinusoidal corrugations. Alasaarela et al. [27] demonstrated polarization-insensitive filtering using a one-dimensional sinusoidal structure in a single grating layer, replacing traditional multilayer dielectric stacks and highlighting the potential of such structures in simplifying device architectures. Sinusoidal gratings are primarily fabricated using holographic interferometry lithography [28], [29], a well-established laser-based interference technique [30], [31]. This method exhibits outstanding manufacturability by directly generating sinusoidal profiles in photoresist through optical interference, thus eliminating the need for complex etching processes. It enables precise control over critical grating parameters, including aspect ratio and period, while supporting the scalable fabrication of large-area, nanoscale periodic structures with high uniformity and reduced production cost.

We propose a design methodology for multicolor-shifting devices with direct orientation tuning, in which simple one-dimensional sinusoidal gratings based on GMR, combined with the proposed evaluation function, enable tailorable color responses, thereby meeting the visual requirements of the human eye across diverse applications. Using this approach, we have successfully designed devices with quad- and penta-color shifting capabilities. Compared to our previous work [32], this paper not only expands the color gamut while preserving high reflectance but also introduces a novel sinusoidal structure. The quad-color shifting device was fabricated, with the master pattern generated via exposed holographic interferometry, where each exposure yields a large-area master mold measuring 3 × 2 cm. Coupled with the low-cost preparation of the master mold and well-established replication processes, this approach provides a practical route toward scalable and cost-effective production, with potential applications in anticounterfeiting and surface decoration. This paper presents the physical model and parameter optimization of the proposed device, followed by tolerance analysis and experimental validation to confirm its practical feasibility.

## 2 Design theory and optimization

Effective medium theory (EMT) is commonly used to describe the macroscopic properties of composite materials or idealized models [33]. When the feature sizes of periodic surface structures are subwavelength relative to the incident wavelength, complex surface structures can be effectively modeled as homogeneous thin films with effective permittivity determined by structural parameters. This paper established an equivalent model for rectangular and sinusoidal gratings. For a rectangular grating with *f* as the duty cycle and *n*_*g*1_ and *n*_*b*1_ as the refractive indices of the grating lines and grating grooves, respectively, the equivalent refractive index of the equivalent material can be obtained:

(1) $$n_{\text{TE}}=\sqrt{\left(1-f\right)n_{b1}^{2}+fn_{g1}^{2}}$$

(2) $$n_{\text{TM}}=\frac{1}{\sqrt{\frac{1-f}{n_{b1}^{2}}+\frac{f}{n_{g1}^{2}}}}$$

For a sinusoidal grating with refractive indices of *n*_*g*2_ and *n*_*b*2_ for the grating lines and grooves, respectively, the equivalent refractive index can be expressed as:

(3) $$n_{\text{TE}}=\sqrt{\frac{n_{b2}^{2}+n_{g2}^{2}}{2}}$$

(4) $$n_{\text{TM}}=\sqrt{\frac{2n_{b2}^{2}n_{g2}^{2}}{n_{b2}^{2}+n_{g2}^{2}}}$$

when the duty cycle *f* of the rectangular grating is 0.5, its effective refractive indices under TE- and TM-polarized illumination match those of equivalent sinusoidal grating structures. This equivalence allows using rectangular gratings as surrogate models for optimization, with derived parameters transferable to sinusoidal counterparts exhibiting similar behavior.

Rotating the subwavelength grating is equivalent to modifying the in-plane component of the incident wavevector, thereby altering the phase-matching condition for exciting guided-mode resonances. This mechanism enables selective high-reflection tuning across different wavelengths by controllably shifting the resonant wavelength of the supported waveguide mode. The device optimization involves multiple parameters, including grating period, groove depth, coating thickness, and multiple azimuthal angles, which together present a vast number of design combinations. Meanwhile, the complex electromagnetic interactions [34] between subwavelength features and incident waves hinder the application of scalar diffraction theory, making it difficult to establish explicit analytical relationships between structural parameters and diffraction efficiencies across various orders. To address these challenges, we employ an immune algorithm characterized by diversity preservation and global search capabilities, integrated with RCWA [35], [36], for accurate vector-field calculations. By defining an evaluation function that weights the diffraction efficiency across the target spectral response range, achieving global optimization of key parameters, ensuring high computational precision, and providing a solid foundation for the inverse design of subwavelength optoelectronic devices.

**Figure 1:**
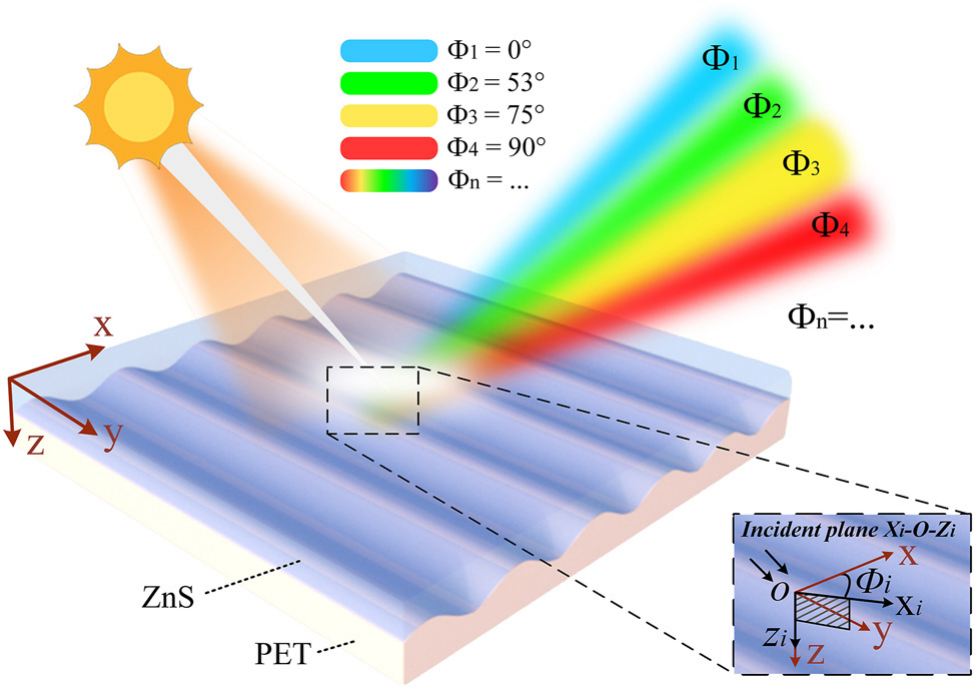
Schematic of the device demonstrating tunable color reflection through in-plane rotation under 45° oblique natural light. Defining the red *o*–*xyz* coordinates in the figure as the reference coordinates, the azimuthal angle Φ*_i_* represents the angle between the incident plane (formed by the incident light direction and the *z*-axis, defined by the *x_i_*–*o*–*z_i_* axes) and the reference *x*–*o*–*z* plane. As the device rotates counterclockwise about the *z*-axis, this angle changes accordingly, reflecting distinct structural colors, for the quad-color device, blue at Φ_1_ = 0°, green at Φ_2_ = 53°, yellow at Φ_3_ = 75°, and red at Φ_4_ = 90°. Multicolor devices share a similar mechanism, additionally offering adjustable azimuthal angles and corresponding colors.

The subwavelength sinusoidal grating discussed herein is designed for illumination under natural light. Figure 1 depicts the physical structure and working principle of the device. The *x*-axis is defined as perpendicular to the grating lines, while the *y*-axis runs parallel to them. The device comprises a single coating layer, a subwavelength grating, and a substrate, all arranged alongside the *z*-axis. The azimuthal angle, denoted as Φ*_i_*, is defined as the angle between the incident plane (formed by the *x_i_*–*o*–*z_i_* axes) and the reference *x*–*o*–*z* plane, where Φ*_i_* = 0° corresponds to the incident plane being perpendicular to the grating lines, that is, the incident *x_i_*–*o*–*z_i_* plane coincides with the reference *x*–*o*–*z* plane. A magnified schematic is provided to aid in understanding the incident plane and the definition of the azimuthal angle Φ*_i_*. As the device rotates about the *z*-axis within its plane, for the quad-color shifting device designed in this work, four distinct structural colors – blue, green, yellow, and red – emerge at azimuthal angles of Φ_1_ = 0°, Φ_2_ = 53°, Φ_3_ = 75°, and Φ_4_ = 90°, respectively. For multicolor shifting devices designed according to this scheme, which respond to other wavelength bands, the azimuthal angle serves as a variable, with the optimal value determined by the optimization algorithm.

**Figure 2:**
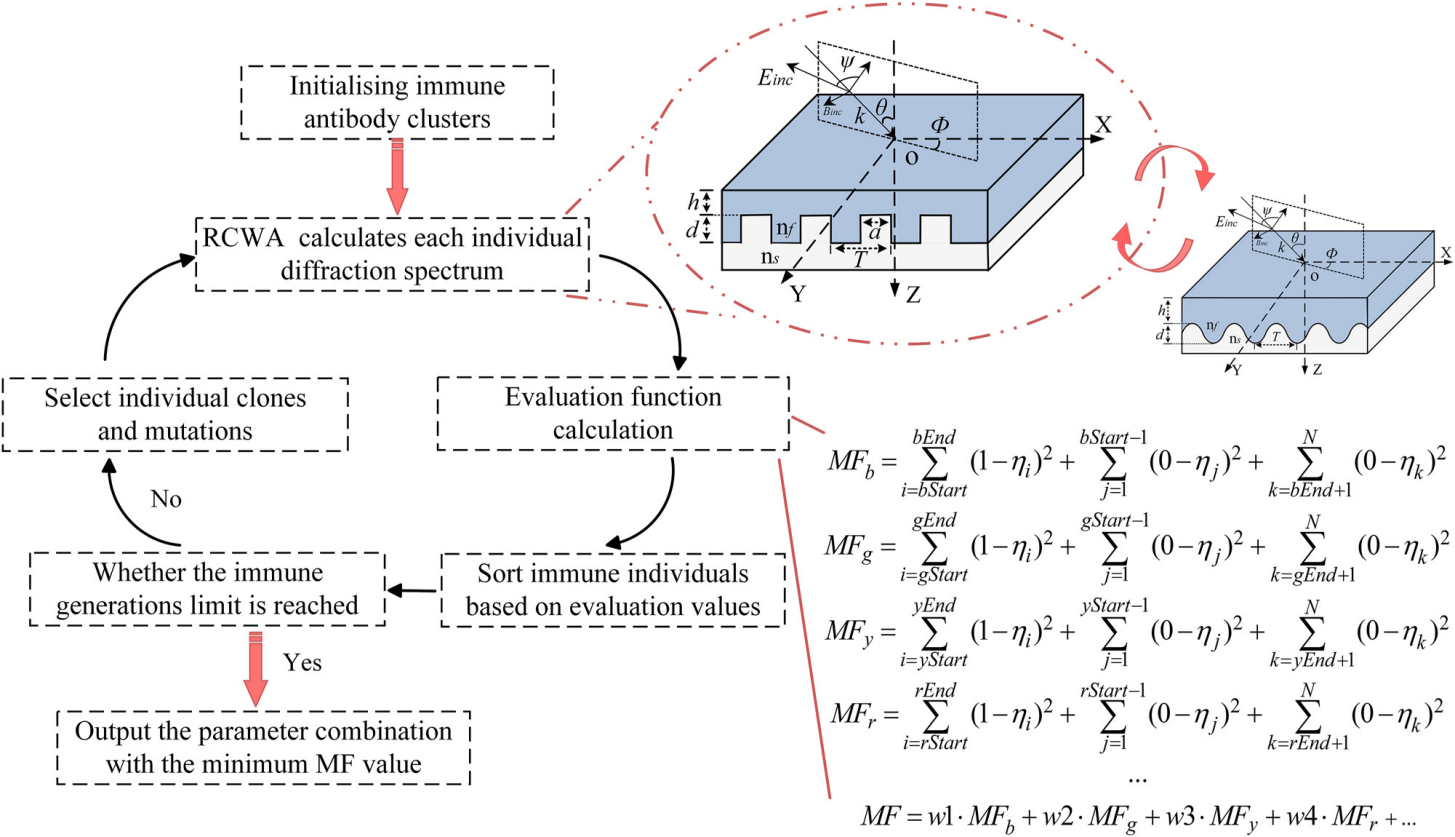
Schematic illustration of the device design flowchart. On the left, the flowchart outlines the device design process, which integrates an immune algorithm with RCWA numerical analysis. The RCWA method analyzes the rectangular structure that is optically equivalent to the proposed sinusoidal structure. The evaluation function for the device’s spectral response is shown in the bottom-right corner of the figure. This function is for a quad-color device, where the core objective is to maximize diffraction efficiency within the target spectral range at a given azimuthal angle while minimizing diffraction efficiency for other spectral ranges. For multicolor devices, the evaluation function can be adjusted accordingly.

This research focuses on the joint optimization of structural parameters and illumination conditions in an angle-dependent structural color device. The evaluation function, denoted as MF, is established to quantify the discrepancy between the diffraction efficiency under natural light and the target spectral response at multiple azimuthal angles. The diffraction efficiency under natural light, *η*, is defined as the average of TE- and TM-polarized components, expressed as *η* = (*η* TE + *η* TM)/2. When designing the quad-color shifting device, initial weights (w1, w2, w3, w4) in the evaluation function are set to 0.25 and are dynamically adjusted during the iterative optimization process. (For the penta-color design, the initial weight is set to 0.2, and for *n*-color designs, the initial weight is set to 1/*n*.) The mathematical form of the evaluation function is shown in Figure 2. Spectral targets are specified for four or more azimuthal angles within the visible range (400–700 nm). For the quad-color design, Azimuth_1 (MFb) requires near-unity reflectance in the blue band with minimal reflection elsewhere. Azimuth_2 (MFg), Azimuth_3 (MFy), and Azimuth_4 (MFr) are designed for high reflectance in the green, yellow, and red bands, respectively, while suppressing spectral crosstalk. Taking the blue azimuthal evaluation function MFb as an example, this represents the function defined for the blue target band (420–460 nm). The device operates within the visible spectrum (400–700 nm). In the actual implementation, *N* denotes the total number of spectral sampling points, calculated as 700 nm − 400 nm = 300 points. The index range for the blue band is defined with bStart = 420 nm − 400 nm = 20, bEnd = 460 nm − 400 nm = 60. Thus, maximizing diffraction efficiency within the indices 20–60 (corresponding to 420–460 nm) while minimizing efficiency across all other sampled wavelengths ensures the minimization of MFb. The same indexing principle applies to the green band, where gStart = 500 nm − 400 nm = 100, gEnd = 540 nm − 400 nm = 140; other colors respond similarly. This evaluation function enables the simultaneous optimization of structural and polarization-dependent optical responses by minimizing MF, ensuring precise spectral alignment with the target color output. Following the formulation of the evaluation function, the design workflow illustrated in Figure 2 is implemented to determine the optimal parameters for the rectangular gratings. The coordinate directions are consistent with those in the previous schematic, although the origin is redefined. The *x*-axis is oriented perpendicular to the grating lines, the *y*-axis runs parallel to them, and the *z*-axis points downward alongside the layer stacking direction. Key structural parameters include the grating period *T*, linewidth *a*, groove depth *d*, duty cycle *f* (*f* = *a*/*T*), and coating thickness *h*. Polyethylene terephthalate (PET, *n*_*s*_ = 1.64) is selected for both the substrate and grating layers due to its widespread use in anticounterfeiting applications. Zinc sulfide (ZnS, *n*_*f*_ = 2.35) is employed as the coating material for its high visible-range refractive index, transparency, and compatibility with PET under vacuum deposition. For incident light with wavelength *λ* and wavevector *k*, the incidence angle *θ* is defined as the angle between *k* and the *z*-axis. The incident plane is spanned by the vectors *k* and *z*. The azimuthal angle Φ*_i_* is set to 0° when the incident plane is perpendicular to the grating lines. The vector polarization angle *ψ* specifies the orientation of the electric field Einc relative to the incident plane, with TE polarization corresponding to *ψ* = 90° and TM polarization to *ψ* = 0°.

## 3 Results and discussions

**Figure 3:**
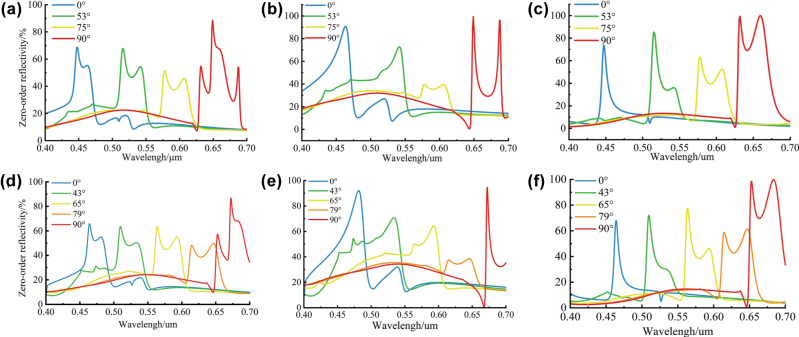
Simulation results. (a) Reflectance spectra under natural light of the quad-color device at four azimuthal angles (0°, 53°, 75°, and 90°). (b) Reflectance spectra for TE-polarized incidence of the quad-color device. (c) Reflectance spectra for TM-polarized incidence of the quad-color device. (d) Reflectance spectra under natural light of the penta-color device at five azimuthal angles (0°, 43°, 65°, 79°, and 90°). (e) Reflectance spectra for TE-polarized incidence of the penta-color device. (f) Reflectance spectra for TM-polarized incidence of the penta-color device.

Based on the physical structure of the device and its target spectral response, and following the previously described design process, multiple rounds of iterative optimization were conducted to identify the optimal parameters. In this process, we designed both quad-color and penta-color shifting devices. Figure 3a–c show the simulated reflection spectra of unpolarized light, TE-polarized light, and TM-polarized light within the visible range (400–700 nm) at an incident angle of 45°, corresponding to azimuthal angles of 0°, 53°, 75°, and 90°, respectively, for the quad-color device. Figure 3d–f, on the other hand, present the simulated reflection spectra of unpolarized, TE-polarized, and TM-polarized within the same wavelength range (400–700 nm) at an incident angle of 45°, corresponding to azimuthal angles of 0°, 43°, 65°, 79°, and 90°, respectively, for the penta-color shifting device. The reflection spectra at each azimuthal angle exhibit distinct resonance peaks, each aligned with the designated target wavelength bands. We present the performance and parameter ranges of the quad-color and penta-color devices in Table 1. Simulation results confirm that the devices achieve wavelength-selective reflection through azimuthal angle control. As the azimuthal angle Φ*_i_* increases from 0° to 90°, the reflection peak shifts progressively across the visible spectrum. Across all azimuthal angles, diffraction efficiencies outside the target bands are effectively suppressed, ensuring high color purity among the four or five primary hues. Minor resonance peak shifts caused by material dispersion have a negligible impact on the observed color. The underlying optical mechanism is beyond the scope of this research and is therefore not discussed here.

**Table 1:** Rectangular grating parameters and peak reflection efficiencies for 45° natural incident light.

Features	Materials	Colors	Azimuths	Targets (nm)	Peaks (nm)	Maximum reflectivity (%)
Quad-color shifting	Coating: ZnS (nf = 2.35)	Blue	0°	420–460	444	67
	Substrate:	Green	53°	500–540	506	68
	PET (ns = 1.64)	Yellow	75°	570–610	574	51
		Red	90°	630–670	641	89
Penta-color shifting	Coating: ZnS (nf = 2.35)	Blue	0°	440–480	469	67
	Substrate:	Green	43°	500–540	511	64
	PET (ns = 1.64)	Yellow	65°	560–600	565	64
		Orange	79°	610–650	644	49
		Red	90°	650–690	678	89

To further validate the feasibility of this equivalence, we performed simulations using device structural units. Table 2 compares the peak positions and diffraction efficiencies of the reflection spectra obtained from the RCWA-optimized rectangular structure, the FDTD rectangular structure, and the FDTD sinusoidal structure. The results show reasonable agreement among all three cases, confirming the accuracy and reliability of the optimization procedure presented in this work and demonstrating that using a sinusoidal grating structure as an equivalent to a rectangular grating is indeed feasible.

**Table 2:** Comparison of reflection spectra metrics under TM incidence obtained from the optimization algorithm and FDTD simulations.

Azimuths	Peak wavelength (nm)			Maximum reflectivity (%)		
	RCWA	FDTD rect	FDTD sine	RCWA	FDTD rect	FDTD sine
0°	444	451	442	73	86	98
53°	508	510	502	82	85	81
75°	574	577	570	63	60	54
90°	626	650	628	100	100	100

**Figure 4:**
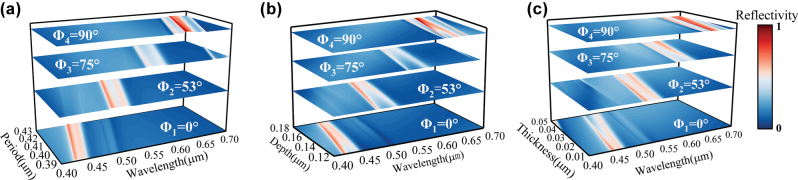
Parameter tolerance. (a) Reflection peak variation with grating period *T* (390 nm–430 nm). (b) Reflection peak variation with groove depth *d* (120 nm–180 nm). (c) Reflection peak variation with coating thickness *h* (10 nm–50 nm). In each figure, the resonant wavelength shift is plotted for all four azimuthal angles. The four curves, arranged from bottom to top, correspond to Φ_1_ through Φ_4_, respectively.

Dimensional deviations in fabrication can lead to mismatches with theoretical optima, causing zeroth-order reflection peak shifts and reduced diffraction efficiency. In this paper, the effects of structural parameter tolerances, namely grating period *T*, groove depth *d*, and coating thickness *h*, on quad-color shifting performance are analyzed through a tolerance analysis. The results quantitatively reveal how variations within defined perturbation ranges influence the spectral positions and efficiency stability of the four target reflection peaks, providing design guidance for tolerance control in nanofabrication. We first examine the tolerance behavior of the grating period *T*. A parameter sweep was conducted around the optimal *T* value, with other parameters fixed at their optimized design values. The scanning range was determined based on the spectral bands of blue, green, yellow, and red light to preserve accurate color rendering at designated azimuthal angles. Under all four azimuthal angles, the reflection peaks show a linear redshift with increasing *T*. Within the range (390 nm, 430 nm), the resonant peak at Φ_1_ = 0° remains in the blue band (420–460 nm), while those at Φ_2_ = 53°, Φ_3_ = 75°, and Φ_4_ = 90° fall into the green, yellow, and red bands, respectively. Thus, maintaining the grating period *T* within (390 nm, 430 nm) ensures stable quad-color shifting performance at the designated azimuthal angles. Applying the same methodology to groove depth *d* and coating thickness *h* shows that, when *d* varies within (120 nm, 180 nm), the characteristic reflection peaks are preserved across all azimuthal orientations. Even when *h* fluctuates over a wider range of (10 nm, 50 nm), the device still maintains its designed blue, green, yellow, and red reflectance bands at Φ_1_ to Φ_4_. Figure 4a–c systematically illustrates the spectral evolution of the reflection peaks under parameter sweeps for *T*, *d*, and *h*, respectively. In particular, Figure 4a shows the resonant wavelength shift versus *T* at Φ_*i*_ = 0°, 53°, 75°, and 90° under a fixed incidence angle of *θ* = 45°, with optimized values of *d* and *h*. Figure 4b and c follow the same structure for *d* and *h* variations.

The redshift in reflection peaks occurs due to altered phase-matching conditions between diffracted fields and leaky waveguide modes. When phase mismatch exceeds a critical threshold, some diffracted energy leaks into transmission channels, reducing resonance peak intensity. To ensure stable quad-color shifting, key structural parameters must stay within *T* = 390–430 nm, *d* = 120–180 nm, and *h* = 10–50 nm, maintaining consistent spectral filtering.

## 4 Fabrication

**Figure 5:**
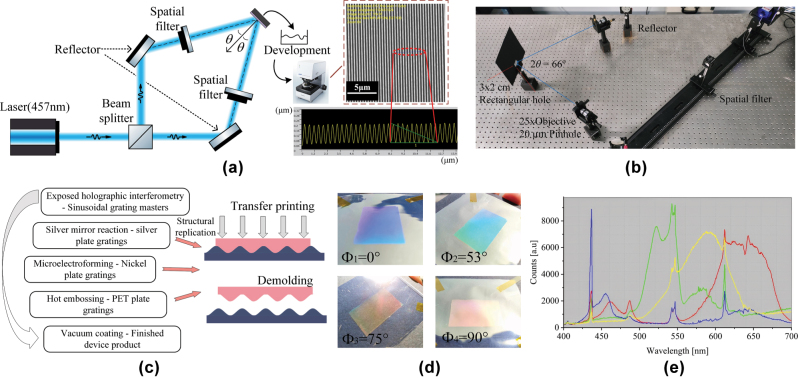
Schemes of the experimental setup and fabricated samples. (a) Experimental setup for sinusoidal grating fabrication via exposed holographic interferometry and the corresponding Olympus laser confocal microscope image of the fabricated structure. (b) Optical layout of the actual experimental system. (c) Subsequent processing steps for sample fabrication from the master template. (d) Appearances of the samples at different azimuthal angles under natural light. (e) Measured reflectance spectra at each azimuthal angle using a fiber-optic spectrometer of a quad-color shifting device.

Using exposed holographic interferometry, a sinusoidal master was fabricated for the quad-color shifting master template, based on EMT and within the allowable fabrication tolerance. The experimental setup employed a 457 nm single-longitudinal-mode solid-state laser as the coherent source, with a beam-splitting system generating two symmetrically incident coherent beams. When the two parallel beams impinge symmetrically at an angle of 2*θ*, their interference field forms a periodic pattern of alternating bright and dark fringes. The resulting fringe period *T* is determined by:

(5) $$T=\frac{\lambda }{2n\sin\theta }$$

where *λ* represents the wavelength of the interference beams, *n* is the refractive index of the propagation medium, and *θ* is the angle between the beam direction and the normal to the interference planes. In the optical setup, the interference angle was set to 2*θ* = 66°, resulting in a theoretical fringe period of *T* ≈ 420 nm, thereby satisfying the fabrication tolerance required by the device design. The depth of the master grating is determined by the combined effects of exposure dose, developer concentration, and development time. Figure 5a and b show the optical path diagram for fabricating the sinusoidal grating master template and the corresponding experimental optical setup, respectively. The three-dimensional morphological characterization of the grating master was conducted using an OLYMPUS laser confocal microscopy system, yielding an average period of 421 nm and a groove depth of 152 nm, falling within the process window defined by theoretical tolerance analysis. Following master template fabrication, electroplating, transfer printing, demolding, and coating were conducted to fabricate the device samples. The process is illustrated in Figure 5c, and the final quad-color sample is presented in Figure 5d. The fabrication described in this section is based on small-scale experiments. For large-scale production, the process would be modified as follows: after creating the nickel master mold, larger substrates can be produced via thermal pressing or UV stitching, followed by silver plating and electroforming to generate large nickel molds. Subsequent embossing and coating steps are performed similarly to the small-scale process, with an expected structural loss of 5–10 % during these steps. Nickel molds, due to their hardness, can support high-volume imprinting, with a single set typically capable of producing 200,000 to 500,000 m of patterned material. Reflectance spectra at different azimuthal angles were measured in real time using a PG2000 spectrometer (Shanghai Ideaoptics Corp., Ltd.). An optical fiber probe was fixed at a 45° angle relative to the device plane, while the sample was rotated horizontally. Spectral data were acquired using Morpho v3.2 spectrometer control software. Figure 5e presents the measured quad-color device reflectance spectra at various azimuthal angles, showing the relationship between reflected intensity and wavelength at different viewing directions. Compared to simulation results, the device exhibits a slight reduction in diffraction efficiency as well as a change in the spectral distribution, with peak wavelength shifts of blue, green, yellow, and red corresponding to 2.70 %, 4.74 %, 1.92 %, and 2.18 %, respectively. Moreover, the spectral widths of the yellow and red channels are significantly broadened, increasing from the designed 40 nm to nearly 75 nm. These deviations are primarily attributed to the following factors: (1) The parameters of the sine grating master plate fabricated using exposed holographic interferometry fall within the redundancy range, but they do not exactly match the optimal values, leading to a reduction in diffraction efficiency; (2) residual developer on the photoresist surface may have continued to react postdevelopment, reducing pattern uniformity; and (3) transfer and coating steps introduced additional alignment and thickness variations. In addition, environmental and human factors during the spectral measurement process may also affect the experimental results. The cumulative effects explain the performance gap between the fabricated and simulated devices. Nevertheless, the samples still exhibit distinct structural colors at the four target azimuthal angles, consistent with the intended optical design.

## 5 Conclusions

In summary, this work proposes an azimuth-controlled multicolor shifting device based on a subwavelength sinusoidal grating. The scheme allows customization of the evaluation function to target the desired color response quantity and range. In this paper, we successfully designed both quad-color and penta-color shifting devices, with expected simulation results. The quad-color device was fabricated, achieving blue, green, yellow, and red color-shifting effects through in-plane rotation. Fabrication of the sinusoidal structure using exposed holographic interferometry enables rapid, scalable production of master molds. We systematically analyzed fabrication tolerances and their influence on the practical filtering performance. With both visual and spectroscopic analysis verification, this approach offers a richer spectral selectivity, simplified fabrication, and reduced costs compared to conventional structural color filtering gratings. After coating, the internal subwavelength structures can no longer be resolved under a microscope; if the coating is damaged, these structures are also destroyed, making it nearly impossible to replicate them, and thus highly suitable for scalable applications such as anticounterfeiting and surface decoration products.
